# Roles of Macrophages and Endothelial Cells and Their Crosstalk in Acute Lung Injury

**DOI:** 10.3390/biomedicines12030632

**Published:** 2024-03-13

**Authors:** Sara Osorio-Valencia, Bisheng Zhou

**Affiliations:** Department of Pharmacology and Regenerative Medicine, University of Illinois College of Medicine, Chicago, IL 60612, USA; sarao@uic.edu

**Keywords:** acute lung injury, macrophages, endothelial cells, inflammation, vascular permeability, lung injury and repair

## Abstract

Acute lung injury (ALI) and its severe form, acute respiratory distress syndrome (ARDS), present life-threatening conditions characterized by inflammation and endothelial injury, leading to increased vascular permeability and lung edema. Key players in the pathogenesis and resolution of ALI are macrophages (Mφs) and endothelial cells (ECs). The crosstalk between these two cell types has emerged as a significant focus for potential therapeutic interventions in ALI. This review provides a brief overview of the roles of Mφs and ECs and their interplay in ALI/ARDS. Moreover, it highlights the significance of investigating perivascular macrophages (PVMs) and immunomodulatory endothelial cells (IMECs) as crucial participants in the Mφ–EC crosstalk. This sheds light on the pathogenesis of ALI and paves the way for innovative treatment approaches.

## 1. Introduction

Acute lung injury (ALI) and its severe manifestation, acute respiratory distress syndrome (ARDS), pose significant threats to life and are commonly associated with critical illnesses such as sepsis, pneumonia, trauma, and, notably, severe cases of COVID-19 [1,2,3,4]. These conditions are characterized by the rapid onset of widespread inflammation and lung damage, leading to severe respiratory failure [2,5,6]. Despite their prevalence and severity, the therapeutic options for ALI and ARDS are still notably limited, underlining an urgent need for research and development in this area [1,5]. The lung’s unique anatomical and physiological characteristics are critical to understanding the pathogenesis of ALI/ARDS. As the most highly vascularized organ, the lung’s extensive network of microvessels is essential for gas exchange and is particularly vulnerable to injury [2,7,8,9,10]. These microvessels are lined by endothelial cells (ECs), which account for about half of all lung cells [7,10,11,12]. ECs are pivotal in maintaining vascular integrity and lung function, regulating processes such as blood vessel tone, blood fluidity, and the barrier function against pathogens and fluid leakage [7,13,14,15,16]. Macrophages (Mφs), the primary immune cells in healthy lung tissue, play a vital role in the lung’s defense mechanism against inhaled pathogens [17,18,19]. They are key players in the immune response, capable of initiating and resolving inflammation [19,20,21,22,23]. The interaction between Mφs and ECs is crucial in the context of ALI/ARDS, where dysregulated inflammation and increased vascular permeability are hallmarks [9,10,13,24,25]. The breakdown in the regulatory mechanisms involving these cells leads to the excessive fluid accumulation, impaired gas exchange, and systemic inflammation characteristic of ALI/ARDS [1,2,5,6,9,10,26,27,28]. Recent research has begun to unravel the complex interplay between Mφs and ECs in the progression and resolution of ALI/ARDS. This body of work suggests that understanding these interactions at a molecular and cellular level could reveal novel therapeutic targets. This review aims to summarize current knowledge on the dynamics between Mφs and ECs within the lung microenvironment during ALI/ARDS and to discuss future research directions that could potentially lead to innovative treatment strategies. The interplay between these cell types is emerging as a critical factor in the disease’s pathogenesis and resolution, offering hope for new approaches to manage and treat these life-threatening conditions.

## 2. Macrophages in Acute Lung Injury

### 2.1. Macrophage Subsets in the Lung

**Figure 1:**
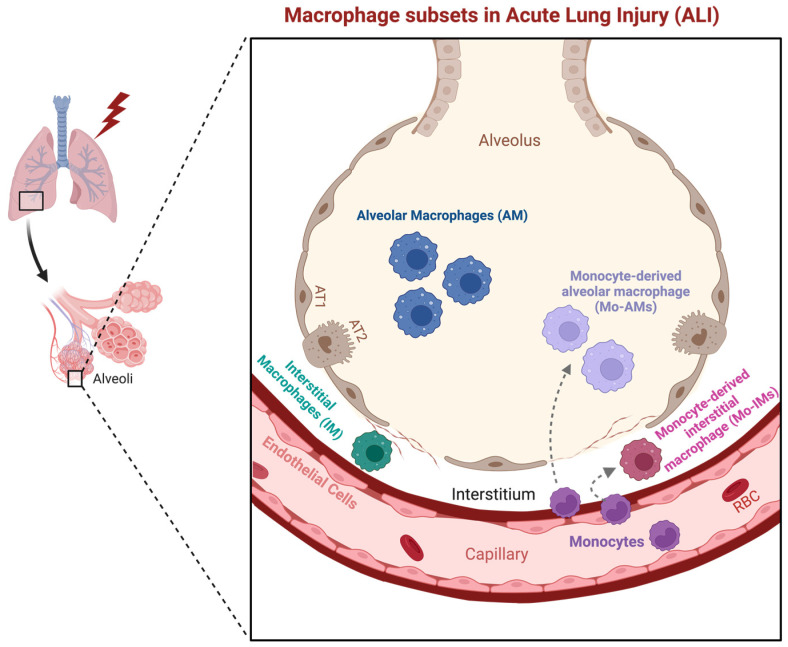
Macrophage subsets in acute lung injury. Two primary resident macrophage populations are present in the lung under normal conditions: alveolar macrophages (AM), located within the alveolar space, and interstitial macrophages (IM), found in the lung interstitium. Following acute lung injury, bone marrow monocytes are recruited through the bloodstream. They can either transmigrate into the interstitial space and differentiate into monocyte-derived interstitial macrophages (Mo-IMs) or enter the alveolar space and differentiate into monocyte-derived alveolar macrophages (Mo-AMs). These distinct macrophage populations play different roles in acute lung injury at various stages. Some are pro-inflammatory, while others are anti-inflammatory or involved in tissue repair. Additionally, these macrophages dynamically interact with the endothelial niche.

In the context of ALI, it is imperative to comprehend the diverse subsets of macrophages inhabiting the pulmonary microenvironment, as they play pivotal roles in both the pathogenesis and resolution of this condition [17,18,29,30,31,32]. The lung harbors distinct populations of macrophages, each endowed with unique anatomical locations and functional attributes [17,22,29,33]. These subsets encompass alveolar macrophages (AMs) [18,34,35], interstitial macrophages (IMs) [36,37,38], and recruited macrophages originating from monocytes (Mo-Mφs) [39,40,41] (Figure 1). AMs reside within the alveolar spaces, IMs are situated in the lung interstitium proximal to the airway epithelium and blood vessels, while Mo-Mφs are recruited in response to pulmonary infections or injuries [17,42]. These subsets have co-evolved with their specific microenvironments, endowing them with tailored features adapted to their respective niches [17,18,43,44,45,46,47].

AMs, as the primary resident macrophages in the alveolar space, act as the frontline defenders against inhaled pathogens and particulate matter [35,48,49]. They exhibit a remarkable proficiency in recognizing and eliminating foreign substances within the airways and alveoli [40,49]. Moreover, they possess the capability to regulate local immune responses through the secretion of cytokines and immune regulatory molecules [34,35,48]. Thus, AMs serve a dual function in immune surveillance and immune modulation, thereby contributing significantly to lung homeostasis.

IMs, positioned within the lung interstitium, serve as key participants in immune surveillance, antigen presentation, and tissue repair processes [36,38,50]. They interact with various immune and structural cell types within the lung microenvironment, thereby orchestrating immune responses and facilitating tissue repair mechanisms [37,38]. These macrophages act as pivotal mediators in coordinating the immune system’s efforts to maintain lung integrity and combat insults to pulmonary health [36,50,51,52].

Mo-Mφs, divided into monocytes-derived interstitial macrophages (Mo-IMs) and monocytes-derived alveolar macrophages (Mo-AMs), play a crucial role in immune surveillance, phagocytosis, cytokine secretion, and antigen presentation [39,40,41,47,53]. Importantly, they are instrumental in regulating inflammation and actively participate in tissue repair processes, thus contributing significantly to the preservation of lung function and health.

In the specific context of ALI, macrophages within the lung microenvironment emerge as central players influencing the inflammatory response, tissue damage, and repair processes [19,22,29,33]. The intricate interplay between macrophage subsets, including AMs, IMs, and recruited Mo-Mφs, is integral to the pathogenesis and resolution of ALI [13,17,21,32,51,54]. Consequently, a comprehensive understanding of the distinct roles and interactions of these macrophage subsets in ALI is imperative for the development of targeted therapeutic strategies aimed at modulating macrophage functions and fostering lung recovery.

### 2.2. Macrophage Polarization in Acute Lung Injury

Macrophages possess remarkable plasticity and demonstrate the ability to adopt distinct functional states, a phenomenon known as macrophage polarization [17,23,55]. These diverse functional states are profoundly influenced by the microenvironmental cues encountered by macrophages [23,29,43,51,56,57]. Broadly, macrophage polarization can be categorized into two primary phenotypes: classically activated (M1) and alternatively activated (M2) states [23,33,55].

M1 macrophages are primarily associated with pro-inflammatory responses and play a pivotal role in host defense against invading pathogens [22,29,54,58,59]. They are characterized by their capacity to produce pro-inflammatory cytokines and generate reactive oxygen species, thereby aiding in the elimination of microorganisms [23,55,60]. In the context of ALI, M1 macrophages are instrumental in initiating the immune response and contributing to tissue damage during the early stages of the condition [17,29,60].

Conversely, M2 macrophages exhibit anti-inflammatory properties and are actively involved in tissue repair processes [21,22,55,61]. They secrete anti-inflammatory cytokines and actively participate in wound healing and tissue remodeling, thus promoting the resolution of ALI and facilitating tissue repair and regeneration [23,33,61]. M2 macrophages are crucial in counteracting excessive inflammation, thereby contributing significantly to the resolution phase of ALI [18,23,33].

The process of macrophage polarization in ALI is dynamic and heavily influenced by various factors present within the lung microenvironment [17,18,30,60,62]. The delicate balance between M1 and M2 polarization states plays a pivotal role in determining the overall inflammatory response and, consequently, the progression or resolution of ALI [31,33,35,55,57]. Therefore, a comprehensive understanding of the mechanisms governing macrophage polarization in the context of ALI is essential for the development of therapeutic strategies aimed at modulating macrophage functions to promote lung healing and recovery.

### 2.3. Macrophage-Derived Soluble Factors in Acute Lung Injury

Macrophages play a central role in the pathogenesis of acute lung injury (ALI) by releasing a diverse array of cytokines, chemokines, and growth factors that intricately regulate immune responses and inflammation within the pulmonary microenvironment [17,22,63].

Cytokines secreted by macrophages, including interleukin-1 beta (IL-1β), tumor necrosis factor-alpha (TNF-α), and interleukin-6 (IL-6), serve as key mediators of the pro-inflammatory response in ALI [63]. These cytokines promote the recruitment and activation of other immune cells, induce dysfunction in endothelial and epithelial cells, and contribute to the disruption of the alveolar–capillary barrier [21,22,58,64,65].

Macrophage-derived chemokines, such as monocyte chemoattractant protein-1 (MCP-1/CCL2), macrophage inflammatory protein-2 (MIP-2/CXCL2), and regulated upon activation, normal T cell expressed and secreted (RANTES/CCL5), play indispensable roles in the recruitment and migration of immune cells to the site of inflammation in ALI [54,56,63,65]. These chemokines amplify and perpetuate the inflammatory response, contributing to the pathological progression of ALI.

Furthermore, macrophages secrete cytokines and growth factors that are vital during the repair and resolution phases of ALI [21,66,67,68]. As they transition from pro-inflammatory to anti-inflammatory states (M1 to M2-like phenotype), macrophages release anti-inflammatory cytokines such as IL-10 and transforming growth factor-beta (TGF-β) [43,66,69]. IL-10 suppresses pro-inflammatory cytokines, aiding in inflammation resolution and tissue repair [66,68,70]. TGF-β stimulates fibroblast and smooth muscle cell proliferation, thus promoting tissue remodeling and angiogenesis [66,70]. Additionally, macrophages secrete growth factors such as platelet-derived growth factor (PDGF) and epidermal growth factor (EGF), further contributing to tissue repair processes. PDGF stimulates fibroblast and smooth muscle cell proliferation, leading to granulation tissue formation, while EGF promotes epithelial cell proliferation and migration, facilitating epithelial repair and regeneration [64,67,70,71].

The intricate network of signaling events orchestrated by the cytokines, chemokines, and growth factors derived from macrophages plays a pivotal role in coordinating immune responses, tissue remodeling, and repair mechanisms during ALI [21,63,68]. Understanding the complex interplay between macrophages, their secreted factors, and other cell types involved in repair processes is indispensable for the development of targeted interventions aimed at enhancing tissue repair and facilitating the resolution of ALI.

### 2.4. Macrophage Inflammasomes and Pyroptosis in Acute Lung Injury

In the context of acute lung injury (ALI), macrophages play a pivotal role, with their function intricately linked to vital mechanisms such as inflammasomes and pyroptosis [6,72,73,74,75,76]. These mechanisms assume particular significance in the pathophysiological processes associated with ALI.

The inflammasome serves as a critical sensor for cellular stress, infection, and inflammation within macrophages [75]. Comprising various proteins, including sensor molecules (NLRP3, NLRC4, or AIM2), an adaptor protein (ASC), and an effector molecule (pro-caspase-1), the inflammasome activation results in the processing and release of pro-inflammatory cytokines, particularly interleukin-1 beta (IL-1β) and interleukin-18 (IL-18) [6,75,76]. Macrophages are major contributors to the production of these cytokines, which play a central role in the inflammatory processes underlying ALI [6,26,74,75].

Pyroptosis, an inflammatory form of cell death, is closely intertwined with inflammasome activation within macrophages [72,73,77,78]. It functions as a defense mechanism, eliminating infected or damaged cells while preventing the spread of pathogens [6,73,75]. Upon inflammasome activation, pro-caspase-1 is cleaved into its active form, caspase-1, subsequently inducing pyroptosis. This process results in the release of pro-inflammatory cytokines, damage-associated molecular patterns (DAMPs), and inflammasome components [73].

In the context of ALI, inflammasome-mediated pyroptosis in macrophages serves multiple functions [6,26,72,73]. It contributes to host defense against infections by facilitating the release of pro-inflammatory cytokines and DAMPs, thereby aiding in the recruitment and activation of other immune cells [6,26,73,74]. Furthermore, macrophage-derived inflammasome activation and pyroptosis play a role in the inflammatory processes underlying lung injury [6,74].

Understanding the intricate interplay between inflammasomes and pyroptosis within macrophages is indispensable for unraveling the mechanisms involved in ALI. Targeting these processes may hold therapeutic potential for mitigating inflammation and effectively managing lung injury, offering promise for improved clinical outcomes in ALI patients.

## 3. Endothelial Cells in Acute Lung Injury

### 3.1. Endothelial Cells (ECs) and Pulmonary Vasculature in Acute Lung Injury

The pulmonary vasculature plays a central role in the lung’s remarkable capacity for gas exchange, a vital physiological process [10,12,14,79]. This capacity is made possible by an intricate network of blood vessels within the lungs, which are lined by specialized ECs [79,80,81,82]. These ECs are particularly abundant in the lung, constituting approximately half of all lung cells [11,15,82,83,84]. This high proportion of ECs provides an extensive surface area that facilitates efficient gas exchange, ensuring the optimal delivery of oxygen to the body [9,14]. Beyond their fundamental role in gas exchange, these ECs play a multifaceted role in the immune system’s response to pathogens and injuries [9,13,24,85].

ECs are integral to the regulation of blood flow, ensuring that oxygen is adequately supplied to the body’s tissues [86,87,88]. Additionally, they are active participants in the immune response, aiding in the recruitment of immune cells to sites of infection or inflammation within the lung [9,89,90]. ECs also contribute to the management of the body’s inflammatory responses, playing a crucial role in maintaining pulmonary homeostasis [79,91].

However, in the context of ALI and its severe form, ARDS, the pulmonary vasculature faces significant challenges. In these pathological conditions, ECs are exposed to various forms of injury that compromise the integrity of the endothelial barrier [1,2,7,10,85]. This disruption leads to an increase in vascular permeability, resulting in the leakage of fluid into the lung tissue, a characteristic feature of pulmonary edema [7,27,83,92,93]. The impairment of this barrier underscores the critical need for therapeutic strategies focused on repairing and regenerating ECs and restoring the integrity of the pulmonary vessels [14,84,94,95,96]. Such interventions hold promise as potential treatments for ALI and ARDS.

### 3.2. Activation and Dysfunction of ECs in ALI/ARDS

The pathogenesis of ALI and ARDS is intricately linked to the activation and dysfunction of ECs within the pulmonary vasculature [8,27,85]. Various triggers, including infections, physical trauma, or inflammatory processes, can activate ECs [10,97,98,99]. Once activated, these cells release pro-inflammatory cytokines, chemokines, and adhesion molecules, which, in turn, facilitate the recruitment and activation of immune cells [85,90,99,100,101]. This cascade of events leads to structural alterations in ECs, ultimately increasing vascular permeability [1,7,16,24]. Consequently, fluid leaks into the lung tissue, contributing to the hallmark feature of pulmonary edema observed in ALI and ARDS [1,14,27,93,102].

Moreover, EC dysfunction plays a disruptive role in the balance between coagulation and fibrinolysis within the lung vasculature [16,27,86,87]. This imbalance leans towards an increased propensity for blood clot formation, further complicating the pathophysiological processes associated with ALI and ARDS [27,86,87,90]. The interplay between EC activation and dysfunction significantly impacts lung function and patient outcomes in these conditions.

### 3.3. Endothelial Regeneration and Vascular Repair in ALI/ARDS

The recovery and resolution of ALI and ARDS heavily depend on the processes of endothelial regeneration and vascular repair [9,11,91,103]. Following an initial insult that damages ECs, these cells initiate a complex process aimed at regenerating and repairing the lung’s blood vessels, with the primary objective of restoring their integrity and functionality [7,12,85,95]. Resident ECs serve as the primary source for this repair process, underscoring the importance of endothelial regeneration in the recovery from ALI and ARDS [16,83,84,93,94,102].

The regenerated ECs play a crucial role in restoring the vascular barrier function, reducing permeability, and preventing the further progression of pulmonary edema. Additionally, they contribute to modulating the inflammatory response by reducing pro-inflammatory signals and increasing the production of anti-inflammatory mediators. Key transcription factors, including FoxM1, Sox17, HIF1α, and COUP-TFII, play significant roles in orchestrating the processes of endothelial regeneration and vascular repair during ALI and ARDS [7,11,83,92,93,94,95,102,104,105]. The concerted efforts of these processes are essential for the recovery of lung function, the resolution of inflammation, and the restoration of vascular integrity.

### 3.4. Lung Endothelial Heterogeneity—Emerging EC Subpopulation and Their Potential Roles in ALI

Recent advancements in single-cell RNA sequencing (scRNA-seq) techniques have unveiled the previously unappreciated heterogeneity of the lung microvascular endothelium [79,81,82,106]. This heterogeneity has identified two distinct populations of capillary ECs with varied roles in lung homeostasis, injury, and repair. These populations include general capillary ECs (gCAPs) and aerocytes (aCAPs), each characterized by unique morphological and functional attributes [80,81].

General capillary ECs (gCAPs) are characterized as thin, flattened cells that function as stem/progenitor cells crucial for endothelial maintenance and repair following injury [79,81,104]. By contrast, aerocytes (aCAPs) are larger, more complex cells located closer to the alveoli, known for their enriched expression of angiogenic factors [81].

These emerging subpopulations of ECs have demonstrated distinct responses to ALI. For instance, aCAPs have been observed congregating within areas of moderate-to-severe alveolar damage in a mouse model of influenza-induced ALI, suggesting their responsiveness to signals from other alveolar cell types [79]. Conversely, a subpopulation of gCAPs, expressing the transcription factor ATF3, demonstrated robust proliferation, contributing to regeneration in the distal lung following similar injuries [104]. These findings indicate a potential regenerative role for gCAPs in lung injury [79,105].

However, despite these intriguing insights, significant gaps remain in our understanding of the specific functions and interactions of these EC subpopulations within the lung, particularly under normal conditions and in the aftermath of ALI. Further research is imperative to elucidate the roles of aCAPs and gCAPs and their potential interactions with other lung cells, such as macrophages, in the context of lung injury and repair. This understanding may hold the key to developing targeted therapeutic strategies to harness the regenerative potential of these EC subpopulations and enhance the recovery process in ALI and ARDS.

## 4. Macrophage–Endothelial Cell Crosstalk in Acute Lung Injury

### 4.1. Macrophage Regulation of Endothelial Function in ALI

The dynamic and multifaceted interplay between Mφs and ECs within the context of ALI constitutes a pivotal axis influencing the pathogenesis, progression, and resolution of this critical pulmonary condition [10,13,17,25,51,107]. Macrophages, serving as the predominant immune cells within the lung parenchyma, exert profound influences on the lung’s endothelium during ALI [13,17,57]. This intricate crosstalk involves a spectrum of interactions, ranging from detrimental effects to beneficial contributions to endothelial homeostasis.

The secretion of various bioactive molecules by macrophages is a cornerstone of their regulatory influence on ECs during ALI. These secreted factors include an array of cytokines, chemokines, and growth factors, which collectively orchestrate EC activation and the modulation of endothelial barrier integrity [17,18,22,42,69,97]. Among these factors, tumor necrosis factor-alpha (TNF-α), interleukin-1 beta (IL-1β), interleukin-6 (IL-6), interferon-gamma (IFN-γ), inducible nitric oxide synthase (iNOS), and reactive oxygen species (ROS) are of particular significance [22,34,63,75,76,97,108]. Binding to specific receptors on ECs, these factors initiate intricate signaling cascades that culminate in the upregulation of adhesion molecules such as vascular cell adhesion molecule-1 (VCAM-1) and intercellular adhesion molecule-1 (ICAM-1) [26,71,97]. This phenomenon promotes the adherence and recruitment of immune cells to sites of inflammation while simultaneously compromising the integrity of the endothelial barrier. Notably, TNF-α-induced matrix metalloproteinase (MMP) production can further exacerbate vascular permeability by degrading the extracellular matrix supporting the endothelial barrier [41,56,109].

Conversely, the later stages of ALI see the emergence of M2 macrophages, characterized by their pro-repair and pro-regeneration properties [21,22,39,48,61,68]. These M2 macrophages release pro-angiogenic factors such as vascular endothelial growth factor (VEGF), fibroblast growth factor 2 (FGF2), interleukin-4 (IL-4), interleukin-10 (IL-10), and transforming growth factor-beta (TGF-β) [33,37,46,61,64,70,110]. These factors play instrumental roles in the regeneration of endothelial cells, thereby facilitating the repair of damaged blood vessels and the restoration of vascular integrity [17,29,41,104]. The dynamic equilibrium between these detrimental and beneficial effects mediated by macrophages is central to the outcome of ALI.

Understanding the intricacies of macrophage regulation of endothelial function in ALI is pivotal for the development of targeted therapeutic interventions. Such interventions aim to mitigate the detrimental effects of excessive inflammation and compromised endothelial barrier function while harnessing the reparative and regenerative potential of M2 macrophages. Ultimately, this knowledge contributes to the broader goal of improving the management and prognosis of ALI.

### 4.2. Endothelial Regulation of Macrophage Function in ALI

**Figure 2:**
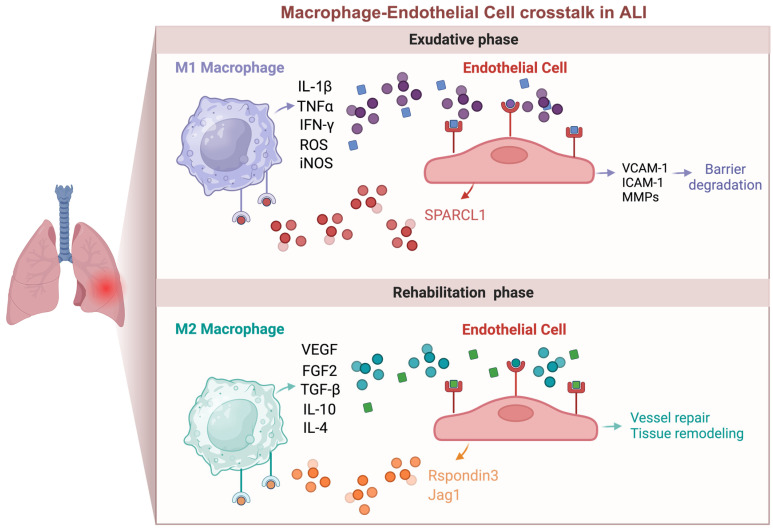
Macrophage and endothelial cell crosstalk in acute lung injury. This interaction is complex and bidirectional, with effects that can be either beneficial or harmful. During the early inflammatory phase of injury, pro-inflammatory macrophages (M1) secrete cytokines like IL-1β and TNF-α, which activate endothelial cells (ECs) and disrupt barrier function. Conversely, activated ECs produce adhesion molecules such as VCAM-1 and ICAM-1, which recruit monocytes and macrophages. In the late phase of injury, ECs can produce angiocrine factors like Rspondin3, which induce macrophages to transition into an anti-inflammatory/pro-repair (M2) state. M2 macrophages, in turn, produce growth factors and cytokines such as VEGF, FGF2, and TGF-β, promoting endothelial regeneration and vascular repair.

The intricate interplay between ECs and macrophages within the pulmonary microvasculature during ALI underscores the pivotal role of ECs in regulating macrophage function and immune responses within the lung [9,14,17,27,42,89]. Lung ECs actively interact with monocytes and macrophages, making ECs central orchestrators of macrophage behavior that influence various aspects such as recruitment, differentiation, renewal, and functional plasticity (Figure 2).

The recruitment of circulating monocytes and their subsequent differentiation into macrophages within the lung tissue are orchestrated, to a significant extent, by ECs [89,103,107,111]. ECs contribute to this process through the production of chemokines like monocyte chemoattractant protein-1 (MCP-1) and the expression of specific receptors like CD31 [24,107]. These molecules are crucial for monocyte recruitment and their differentiation into macrophages, highlighting the role of ECs as gatekeepers in modulating the immune cell population within the lung microenvironment [9,41,89,107].

Furthermore, ECs profoundly impact macrophage polarization, a key determinant of the nature of the immune response [13,14,89]. Through the secretion of soluble factors, ECs influence the macrophage phenotype, tipping the balance between pro-inflammatory M1 and anti-inflammatory M2 macrophages [51,98,100,111,112]. This polarization has far-reaching consequences for the progression or resolution of ALI. For instance, the secretion of interleukin-4 (IL-4), interleukin-10 (IL-10), and transforming growth factor beta (TGF-β) by ECs promotes the expansion of M2 macrophages, thereby eliciting anti-inflammatory responses [61,68,100]. These M2 macrophages express surface markers like CD206 and produce increased levels of IL-10, contributing to inflammation resolution and endothelial repair [13,14,15,68].

Beyond these soluble mediators, specific interactions between ECs and macrophages underscore the regulatory role of ECs in modulating macrophage function during ALI [13,25,101]. For instance, lung ECs release Rspondin3, an angiocrine factor that activates the Wnt/β-catenin signaling pathway in interstitial macrophages [13,51]. This activation initiates a metabolic–epigenetic reprogramming of macrophages, promoting anti-inflammatory responses and endothelial repair [13,51].

Moreover, the influence of ECs extends to the regulation of alveolar macrophages through endothelial-derived exosomes, which target RGS1-mediated calcium signaling-dependent immune responses [113]. These mechanisms result in improved outcomes in ALI/ARDS [113]. Additionally, endothelial Jagged1 (Jag1) has been identified as a regulator of macrophage polarization, preventing radiation-induced lung injury through Notch signaling [100]. Also, certain cytokines produced by lung ECs can induce a pro-inflammatory phenotype transition in macrophages. For instance, SPARCL1 derived from ECs exacerbates viral pneumonia by activating pro-inflammatory macrophages [98].

In summary, the intricate and bidirectional interactions between ECs and macrophages in ALI highlight the pivotal role of ECs in shaping macrophage behavior and inflammatory responses [13,25,89]. Understanding these complex regulatory mechanisms is crucial for the development of targeted therapeutic interventions that aim to modulate macrophage function through endothelial regulation, ultimately leading to improved outcomes and enhanced lung repair in ALI. Further research in this area holds great promise for advancing our understanding of ALI pathogenesis and optimizing clinical management strategies.

## 5. Future Perspectives

### 5.1. Perivascular Macrophages (PVMs)

Perivascular macrophages (PVMs) are a unique subset of interstitial macrophages that inhabit the perivascular spaces of various tissues, including the lungs [50,114]. These specialized macrophages occupy a strategic position at the interface between the bloodstream and surrounding tissue, allowing them to perform crucial functions related to vascular homeostasis, immune surveillance, and tissue repair [114,115,116,117,118,119,120,121,122,123].

In the context of ALI, the specific roles and mechanisms of PVMs remain relatively uncharted territory in scientific research. However, their distinctive location within the vascular niche suggests that they may play a significant role in supporting lung vascular integrity and participating in vascular repair following injury [50,114,116,124,125]. To gain a deeper understanding of PVMs’ contributions to ALI, it is essential to delve into their potential functions and their interactions with other immune and endothelial cell types.

PVMs, like other macrophage populations, are equipped with the ability to phagocytose pathogens, debris, and cellular waste [47,50,114,122,123,126]. In the context of ALI, where the lung is subjected to various insults such as infections, trauma, or inflammatory processes, PVMs are likely to participate in the clearance of foreign invaders and damaged cellular components [45,47,50]. This phagocytic activity is crucial for limiting the extent of tissue damage and initiating the repair process.

Furthermore, PVMs may possess antigen-presenting capabilities, similar to dendritic cells, which can play a pivotal role in immune responses [114,119,127]. In ALI, antigens from pathogens or damaged tissue may be processed and presented by PVMs, thus contributing to the recruitment and activation of other immune cells. This interaction is critical for mounting an effective immune response against the underlying cause of ALI.

One intriguing aspect of PVMs’ potential function in ALI is their role in maintaining immune tolerance. These macrophages are strategically positioned in close proximity to the bloodstream and can serve as gatekeepers, regulating the entry of immune cells and molecules into the lung tissue [114,116,125]. In the context of ALI, where immune dysregulation and excessive inflammation can exacerbate tissue damage, PVMs may exert an immunomodulatory role by tempering immune responses and preventing the excessive infiltration of immune cells.

PVMs are also likely to be involved in the regulation of endothelial barrier function within the lung microvasculature. Given their proximity to blood vessels, these macrophages may participate in the maintenance of vascular integrity and the repair of damaged vessels [47,114,116,124]. In conditions such as ALI, where increased vascular permeability and leakage of fluid into lung tissue are characteristic features, understanding how PVMs contribute to endothelial barrier function and repair is of paramount importance.

Research efforts aimed at unraveling the functions of PVMs in ALI should include investigations into their interactions with other immune cells, particularly macrophages of different subsets, as well as endothelial cells [114,118,120]. It is conceivable that PVMs play a role in coordinating the immune response by influencing the recruitment and activation of immune cells, such as neutrophils, monocytes, and lymphocytes, which are known to be involved in ALI. Additionally, PVMs may engage in bidirectional crosstalk with endothelial cells, contributing to the regulation of vascular permeability and tissue repair processes [114,123,126].

Potential therapeutic strategies targeting PVMs in ALI may involve modulating their phagocytic activity, enhancing their antigen-presenting capabilities, or manipulating their immunomodulatory properties [114,123]. These approaches could be designed to harness the protective functions of PVMs while minimizing the detrimental effects of excessive inflammation.

In conclusion, while the roles of PVMs in ALI remain largely unexplored, their strategic location in the vascular niche and their potential functions in phagocytosis, antigen presentation, immune regulation, and vascular maintenance make them intriguing candidates for further investigation. Understanding the contributions of PVMs to ALI pathogenesis and resolution may pave the way for innovative therapeutic approaches aimed at improving patient outcomes in this devastating condition.

### 5.2. Immunomodulatory Endothelial Cells (IMECs)

ECs are well recognized for their pivotal role in regulating inflammation, primarily by controlling the trafficking, activation, and function of immune cells, including macrophages [13,16,86,89,101,128]. Recent advancements in single-cell analysis techniques have unveiled the existence of specific subtypes of ECs with distinct immunomodulatory capacities in various tissues and organs, including the lungs [80,82,101,104,106]. These specialized EC subsets, collectively referred to as immunomodulatory endothelial cells (IMECs), exhibit unique properties that enable them to interact with immune cells, particularly macrophages, influencing their recruitment, activation, and regulation [89,101]. IMECs represent a paradigm shift in our understanding of EC diversity and function within the context of immune responses. In ALI, a condition characterized by dysregulated inflammation and immune cell infiltration, IMECs have emerged as key players in shaping the immune landscape of the lungs and influencing the course of the disease [13,82,89].

One of the critical functions attributed to IMECs in ALI is their role in immune cell recruitment. IMECs are capable of producing chemokines and adhesion molecules that facilitate the recruitment of immune cells to sites of inflammation [89,101]. By modulating the expression of these molecules, IMECs can orchestrate the trafficking of immune cells, including macrophages, to the injured lung tissue. This process is essential for mounting an effective immune response against pathogens or damage-associated molecular patterns (DAMPs) in ALI.

Moreover, IMECs are believed to participate in the regulation of immune cell activation and function [89,101]. By secreting soluble factors, such as cytokines and growth factors, IMECs can influence the polarization of macrophages, determining whether they adopt a pro-inflammatory (M1) or anti-inflammatory (M2) phenotype. This polarization state of macrophages significantly impacts the inflammatory response and tissue repair processes in ALI. IMECs may act as immune modulators, skewing the balance between pro-inflammatory and anti-inflammatory responses within the lung microenvironment [101].

Recent studies have identified specific subpopulations of ECs, such as “immuneECs”, which exhibit a heightened inflammatory response upon exposure to pathogens [82]. These specialized ECs may serve as vanguards in activating host defense mechanisms in response to airborne pathogens like SARS-CoV-2 or the influenza virus [82]. Understanding the activation mechanisms and functional properties of immuneECs and similar EC subsets is essential for deciphering their contributions to ALI pathogenesis.

IMECs also possess the capacity to produce molecules that can either enhance or suppress immune cell activity [101]. This dual role in immune modulation positions IMECs as central players in fine-tuning the immune response during ALI. By elucidating the signaling pathways and molecules involved in IMEC-mediated immune regulation, novel therapeutic approaches can be developed to modulate the immunomodulatory properties of these ECs and restore immune balance in ALI.

In conclusion, research focused on IMECs within the context of ALI represents a cutting-edge field with significant therapeutic potential. These specialized EC subsets are poised to play crucial roles in regulating immune responses, immune cell recruitment, and immune cell activation in ALI. By deciphering their functions, signaling pathways, and contributions to lung inflammation and repair, innovative strategies can be developed to harness the immunomodulatory properties of IMECs for the benefit of patients with ALI. These interventions may involve the targeted manipulation of IMECs to enhance their protective functions and mitigate the detrimental effects of excessive inflammation, ultimately improving the prognosis of ALI.

### 5.3. Future Perspectives and Therapeutic Opportunities

The crosstalk between macrophages and ECs represents a complex and dynamic interplay that underlies the pathogenesis and resolution of ALI. Understanding the intricate interactions between these two cell types is of paramount importance for the development of targeted therapies aimed at modulating the inflammatory response and promoting tissue repair in ALI. The exploration of PVMs and IMECs within this context offers exciting opportunities for future research and therapeutic interventions.

As the scientific community delves deeper into the functions of PVMs, it is crucial to uncover their specific roles in ALI pathogenesis and resolution. Investigating their interactions with other immune cells, such as neutrophils, monocytes, and lymphocytes, can provide valuable insights into the orchestration of immune responses during ALI. Additionally, understanding how PVMs influence endothelial barrier function and vascular repair processes may open new avenues for therapeutic strategies targeting vascular integrity and immune regulation in ALI.

Therapeutic approaches targeting PVMs could involve modulating their phagocytic activity, enhancing their antigen-presenting capabilities, or fine-tuning their immunomodulatory properties. By harnessing the protective functions of PVMs and mitigating excessive inflammation, these interventions have the potential to improve patient outcomes and enhance our ability to combat ALI.

On the other front, the exploration of IMECs and their diverse roles in immune cell recruitment, activation, and regulation holds great promise for advancing our understanding of ALI. Research efforts should focus on deciphering the signaling pathways and molecules involved in IMEC-mediated immune modulation. This knowledge can be leveraged to develop innovative therapeutic approaches that modulate the immunomodulatory properties of IMECs and restore immune balance in ALI.

Therapeutic strategies targeting IMECs may encompass the manipulation of chemokines, cytokines, and growth factors produced by these EC subsets. By influencing the recruitment, activation, and polarization of immune cells, including macrophages, these interventions can rebalance the immune response and promote tissue repair in ALI.

In the broader context of ALI research, recent advances in single-cell spatial biology have facilitated the discovery of previously unknown cell types and subpopulations. This includes the identification of PVMs and IMECs, which are likely to have critical roles in the crosstalk between macrophages and endothelial cells in ALI. Future studies should continue to explore these newly identified cell populations, uncover their functions, and elucidate their contributions to disease pathogenesis and resolution.

Moreover, the translation of these promising research findings into clinical practice represents a significant challenge and opportunity. Therapeutic interventions targeting PVMs and IMECs must undergo rigorous preclinical and clinical evaluation to ensure their safety and efficacy in ALI patients. Developing innovative therapeutic modalities, such as monoclonal antibodies or small molecule inhibitors, that specifically target PVMs or IMECs may hold the key to successful clinical translation.

In summary, the future of ALI research and therapy lies in our ability to unravel the complexities of macrophage–endothelial cell crosstalk. PVMs and IMECs represent novel players in this intricate interplay and offer exciting prospects for therapeutic interventions. By understanding their functions, signaling pathways, and contributions to lung inflammation and repair, we can develop targeted strategies to modulate their activity and restore immune balance in ALI. Continued research in this area is imperative for advancing our knowledge and improving the prognosis of patients with this devastating condition.
